# Human pregnancy-associated malaria-specific B cells target polymorphic, conformational epitopes in VAR2CSA

**DOI:** 10.1111/j.1365-2958.2006.05503.x

**Published:** 2007-01

**Authors:** Lea Barfod, Nadia L Bernasconi, Madeleine Dahlbäck, David Jarrossay, Pernille Haste Andersen, Ali Salanti, Michael F Ofori, Louise Turner, Mafalda Resende, Morten A Nielsen, Thor G Theander, Federica Sallusto, Antonio Lanzavecchia, Lars Hviid

**Affiliations:** 1 Centre for Medical Parasitology at Department of Infectious Diseases, Copenhagen University Hospital (Rigshospitalet) and Institute for Medical Microbiology and Immunology, University of CopenhagenCopenhagen, Denmark.; 2 Institute for Research in BiomedicineBellinzona, Switzerland.; 3 Center for Biological Sequence Analysis, BioCentrum-DTU, Technical University of DenmarkLyngby, Denmark.; 4 Department of Immunology, Noguchi Memorial Institute for Medical Research, University of GhanaLegon, Ghana.

## Abstract

Pregnancy-associated malaria (PAM) is caused by *Plasmodium falciparum*-infected erythrocytes (IEs) that bind to chondroitin sulphate A (CSA) in the placenta by PAM-associated clonally variant surface antigens (VSA). Pregnancy-specific VSA (VSA_PAM_), which include the PfEMP1 variant VAR2CSA, are targets of IgG-mediated protective immunity to PAM. Here, we report an investigation of the specificity of naturally acquired immunity to PAM, using eight human monoclonal IgG1 antibodies that react exclusively with intact CSA-adhering IEs expressing VSA_PAM_. Four reacted in Western blotting with high-molecular-weight (> 200 kDa) proteins, while seven reacted with either the DBL3-X or the DBL5-ε domains of VAR2CSA expressed either as *Baculovirus* constructs or on the surface of transfected Jurkat cells. We used a panel of recombinant antigens representing DBL3-X domains from *P. falciparum* field isolates to evaluate B-cell epitope diversity among parasite isolates, and identified the binding site of one monoclonal antibody using a chimeric DBL3-X construct. Our findings show that there is a high-frequency memory response to VSA_PAM_, indicating that VAR2CSA is a primary target of naturally acquired PAM-specific protective immunity, and demonstrate the value of human monoclonal antibodies and conformationally intact recombinant antigens in VSA characterization.

## Introduction

People living in areas of intense transmission of *Plasmodium falciparum* parasites acquire protective immunity to malaria during childhood, and the bulk of mortality and severe morbidity from *P. falciparum* malaria is therefore concentrated among young children. Protective immunity acquired in response to *P. falciparum* exposure appears to be mediated mainly by IgG antibodies specific for variant surface antigens (VSA) that mediate sequestration of infected erythrocytes (IEs) in various tissues (reviewed by Hviid, 2005). Despite pre-existing protective immunity, women become highly susceptible to *P. falciparum* infection when they become pregnant, and pregnancy-associated malaria (PAM) is a major cause of mother/offspring morbidity (Guyatt and Snow, 2001; 2004). However, in areas of stable *P. falciparum* transmission, susceptibility to PAM rapidly declines with increasing parity, consistent with acquisition of PAM-specific protective immunity (reviewed by Hviid, 2004). PAM is caused by *P. falciparum*-IEs selectively accumulating in the placental intervillous space through VSA_PAM_-mediated adhesion to chondroitin sulphate A (CSA). VSA_PAM_ differ in several ways from VSA expressed on IEs obtained from males and non-pregnant females. Thus, only VSA_PAM_ mediate binding to CSA *in vitro* (Fried and Duffy, 1996) and only VSA_PAM_-expressing IEs are consistently not recognized by IgG in the plasma of *P. falciparum*-exposed women who have never been pregnant or by IgG in plasma from similarly exposed men (Beeson *et al*., 1999; Ricke *et al*., 2000). These observations, and the fact that plasma levels of VSA_PAM_-specific IgG increase with increasing parity (Fried *et al*., 1998; Ricke *et al*., 2000), are consistent with evidence that these antibodies are the mediators of protective immunity to PAM (Duffy and Fried, 2003; Staalsoe *et al*., 2004).

The molecular identity of VSA_PAM_ remains controversial, although current evidence points to VAR2CSA, an interclonally conserved member of the PfEMP1 molecules encoded by the multigene *var* family. Thus, transcription of the gene encoding VAR2CSA is increased among CSA-adhering and placental isolates, VAR2CSA is exposed on the surface of CSA-adhering IEs (Salanti *et al*., 2003; 2004; Tuikue Ndam *et al*., 2005), and plasma levels of VAR2CSA-specific IgG increase with increasing parity and correlate with protective immunity to PAM (Salanti *et al*., 2004). However, the importance of VAR2CSA-specific antibodies relative to antibodies specific for other putative VSA_PAM_ in acquired protective immunity to PAM remains to be established. The clonal analysis of memory B cells represents a powerful tool to dissect the immune response to complex pathogens such as *P. falciparum* (Lanzavecchia *et al*., 2006). In this study, we used an improved Epstein–Barr virus (EBV) immortalization method (Traggiai *et al*., 2004) to analyse memory B cells from multiparous PAM-exposed women. Frequency analysis and isolation of specific monoclonal antibodies identified polymorphic, linear and conformation-dependent epitopes in VAR2CSA as dominant targets of the human memory B-cell response to PAM.

## Results and discussion

### PAM induces a high-frequency VSA_PAM_-specific memory B-cell response

**Fig. 1:**
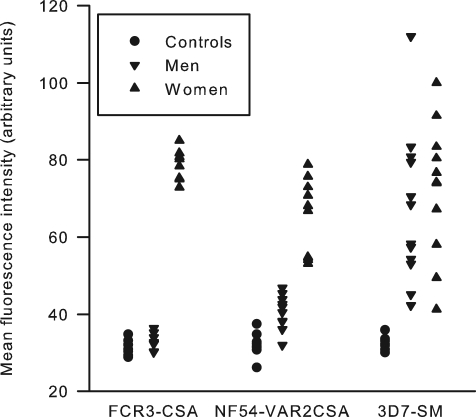
Flow cytometry analysis of human VSA-specific plasma IgG reactivity with the surface of *P. falciparum*-IEs. Labelling of FCR3-CSA, NF54-VAR2CSA and 3D7-SM by IgG in individual plasma samples from *P. falciparum*-exposed pregnant women (▴), from sympatric men (▾) and from non-exposed adult control donors (•) are shown.

We first used flow cytometry to screen plasma from 27 PAM-exposed and recently pregnant multigravidae for IgG antibodies capable of staining *P. falciparum*-IEs expressing VSA_PAM_ (Staalsoe *et al*., 1999; Ricke *et al*., 2000). We selected three donors (one parity 2 and two parity 3 women) with high VSA_PAM_-specific plasma antibody levels and used frozen peripheral blood mononuclear cells (PBMC) obtained 1 month post-partum. Memory B cells were immortalized with EBV in the presence of CpG oligonucleotides and allogeneic, irradiated PBMC as described (Traggiai *et al*., 2004). A total of 5760 replicate cultures of 100 immortalized B cells per well were set up, and after 3 weeks the culture supernatants were screened for their capacity to stain erythrocytes infected with each of three *P. falciparum* lines. Two of the lines (FCR3-CSA and NF54-VAR2CSA) had been previously selected *in vitro* to express VSA_PAM_, characterized by reactivity with IgG from multiparous women and lack of reactivity with IgG from *P. falciparum*-exposed men (Fig. 1) (Fried *et al*., 1998; Beeson *et al*., 1999; Ricke *et al*., 2000). The third line (3D7-SM) was selected to express non-PAM-type VSA equally recognized by IgG from *P. falciparum*-exposed men and women (Fig. 1) (Staalsoe *et al*., 2003; Jensen *et al*., 2004). Supernatants from 105 of the polyclonal B-cell lines stained one or both of the VSA_PAM_-expressing lines. The frequency of VSA_PAM_-reactive polyclonal supernatants varied from 6/1920 [0.3% (95% confidence interval: 0.1–0.7%)] to 33/1344 [2.5% (1.8–3.4%)] in the three donors. These results suggest that the frequency of VSA_PAM_-specific B cells can be high (at least up to 1 in 4000 memory B cells) in recently pregnant multigravidae. The higher memory B-cell frequencies in the present study compared with earlier reports for PfEMP1 (Dorfman *et al*., 2005) and total *P. falciparum* antigens (Fievet *et al*., 1993; Migot *et al*., 1995) probably reflect the efficient method of B-cell immortalization employed here.

### VSA_PAM_-specific human monoclonal IgG1 antibodies specifically recognize polymorphic epitopes on IEs selected for adhesion to CSA

**Fig. 2:**
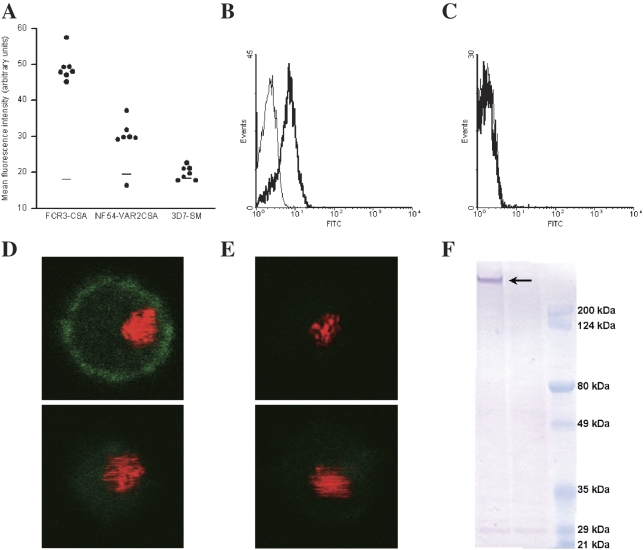
Reactivity of human IgG monoclonal antibodies with *P. falciparum*-IEs. A. Labelling of FCR3-CSA, NF54-VAR2CSA and 3D7-SM by VSA_PAM_-specific monoclonal antibodies (•) or an irrelevant control monoclonal antibody (–), determined by flow cytometry. B. Reactivity of monoclonal antibody PAM3.10 (heavy line) and an irrelevant control monoclonal antibody (thin line) with the surface of erythrocytes infected by FCR3-CSA. C. PAM3.10 reactivity with the surface of erythrocytes infected by unselected FCR3. D. Immunofluorescence microscopy of FCR3-CSA-infected erythrocytes labelled with PAM3.10 (top) or an irrelevant control antibody (bottom). E. Immunofluorescence microscopy of unselected FCR3-IEs labelled with PAM3.10 (top) or an irrelevant control antibody (bottom). F. Reactivity of PAM3.10 in Western blots of FCR3-CSA (left) and FCR3 (centre). Broad-range molecular weight markers are shown in the right lane.

Cloning of EBV-immortalized IgG^+^ B cells from 28 of the VSA_PAM_-specific lines by limiting dilution resulted in eight clones producing VSA_PAM_-specific IgG1. Lines were selected for cloning on the basis of their IgG synthesis and growth characteristics. Six of the clones (PAM1.4, PAM2.8, PAM3.10, PAM5.2, PAM6.1, PAM7.5) produced antibodies recognizing antigens on the surface of erythrocytes infected by both the VSA_PAM_-expressing lines used to screen for antibody specificity (Table 1). Antibodies from the two remaining clones (PAM4.7 and PAM8.1) only recognized FCR3-CSA. In contrast, none of the monoclonal antibodies recognized the 3D7-SM control line not expressing VSA_PAM_ (Fig. 2A–C). Testing of monoclonal antibody reactivity with erythrocytes infected by a panel of additional parasite lines provided further evidence that all were indeed specific for PAM-type VSA expressed on the surface of CSA-adhering IEs (Table 1). However, the monoclonal antibodies did not all recognize all VSA_PAM_-expressing lines, probably because the epitopes they recognize are polymorphic. IgG antibodies produced by a control B-cell clone (D7) did not recognize any of the tested parasite lines. Monoclonal antibody recognition patterns for individual parasite lines were tested in parallel, and repeated assessments of recognition patterns yielded consistent results.

**Table 1:** Reactivity of human monoclonal IgG1 antibodies with the surface of erythrocytes infected by parasite lines, determined by flow cytometry.

			Monoclonal antibody								
Parasite line	VSA_PAM_ expression^a^	IE adhesion to CSA	PAM1.4	PAM2.8	PAM3.10	PAM4.7	PAM5.2	PAM6.1	PAM7.5	PAM8.1	D7
3D7-BeWo^b^	+	+/–	+	+	+	–	+	+	+	–	–
3D7-SM^c^	–	–	–	–	–	–	–	–	–	–	–
EJ24	–	–	–	–	–	–	–	–	–	n.d.	–
EJ24-PAM1.4	+	n.d.	+	+	+	–	–	+	+	n.d.	–
EJ27	–	–	–	–	–	–	–	–	–	n.d.	–
EJ27-PAM1.4	+	n.d.	+	–	–	–	–	–	–	n.d.	–
FCR3-A745	–	–	–	–	–	–	–	–	–	–	–
FCR3-CD36	–	–	–	–	–	–	–	–	–	–	–
FCR3-CSA^c^	+	+	+	+	+	+	+	+	+	+	–
NF54^b^	–	–	–	–	–	–	–	–	–	–	–
NF54-VAR2CSA^c^	+	+	+	+	+	–	+	+	+	–	–

a All VSA_PAM_^+^ lines transcribed *var2csa* (data not shown). See Fig. 1 and *Experimental procedures* for definition of VSA_PAM_ expression.

b 3D7 (Walliker *et al*., 1987) was originally cloned from, and appears genetically identical to, NF54 (Delemarre and Van der Kaay, 1979).

c Line used in screening of B-cell supernatants for production of VSA_PAM_-specific IgG.

n.d., not determined.

The flow cytometry evidence of antibody reactivity with antigens on the surface of IEs expressing VSA_PAM_ and the absence of reactivity with non-PAM-type VSA (Table 1) was confirmed by immunofluorescence microscopy of live IEs (Fig. 2D and E). Denaturing Western blots of the VSA_PAM_-expressing sublines yielded single, distinct bands (of similar size for each antibody) when probed with PAM3.10, PAM5.2, PAM6.1 and PAM7.5 monoclonal antibodies (Fig. 2F, and data not shown). Proteins were not detected when blots were probed with the monoclonal antibodies PAM1.4, PAM2.8 or PAM4.7 (data not shown) despite their reactivity with the surface of intact VSA_PAM_-expressing IEs (Table 1), pointing to reactivity with conformation-dependent epitopes. No bands were observed when the monoclonal antibodies were used to probe Western blots of the non-PAM-type VSA-expressing parental lines (Fig. 2F, and data not shown). PAM8.1 was not tested by Western blotting with IEs expressing VSA_PAM_, but was tested with VAR2CSA-specific constructs (see below).

### VAR2CSA is a dominant target of the human immune response to pregnancy-associated malaria

**Fig. 3:**
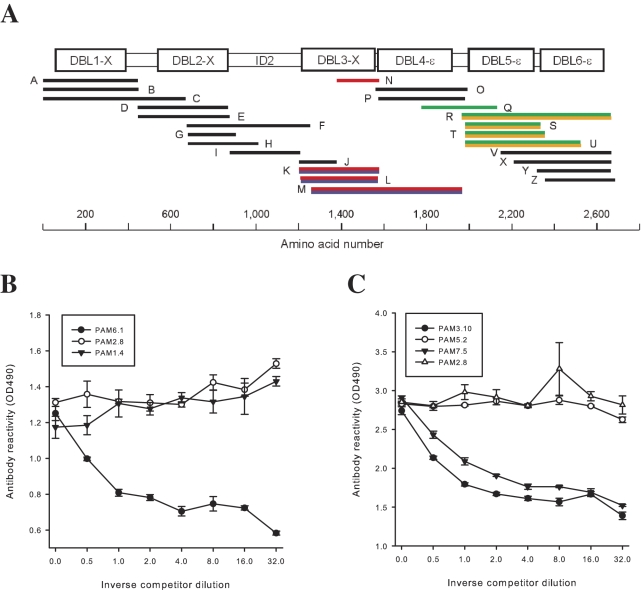
Reactivity of human VSA_PAM_-specific IgG1 monoclonal antibodies with VAR2CSA. A. The top panel shows a schematic representation of VAR2CSA with the positions of the recombinant protein constructs used (A–Z) and the amino acid numbers indicated along the bottom. Recognition of constructs A, E, I, L, P, T and Z in ELISA, and flow cytometric recognition of Jurkat cells transfected to express constructs B–D, F–H, J, K, M–O, Q–S, U–Y by antibodies PAM2.8 (red), PAM3.10 (orange), PAM5.2 and PAM7.5 (green), and PAM6.1 (blue) are shown. B. PAM6.1-specific competition ELISA to determine domain specificity of DBL3-X-reactive IgG (competitors shown in the figure). C. PAM3.10-specific competition ELISA to determine domain specificity of DBL5-ε-reactive IgG (competitors shown in the figure).

The high molecular weight of the proteins detected by Western blotting (Fig. 2F) suggested that the monoclonal antibodies were specific for members of the so far best-characterized family of VSA, PfEMP1 (Leech *et al*., 1984). This family includes VAR2CSA (predicted molecular weight: 355 kDa), which is the only PfEMP1 described so far that has the characteristics expected of VSA_PAM_ (Salanti *et al*., 2003; 2004). We therefore used a panel of recombinant proteins spanning the entire extracellular part of VAR2CSA from 3D7 (Fig. 3A) and FCR3 (data not shown) to examine the antigen specificity of the monoclonal VSA_PAM_-specific IgG antibodies further. Antibodies PAM2.8, PAM3.10, PAM5.2, PAM6.1 and PAM7.5 tested positive in 3D7-VAR2CSA domain-specific ELISA (Fig. 3A and Table 2), while antibodies PAM2.8, PAM3.10, PAM4.7, PAM5.2 and PAM8.1 tested positive in the FCR3-VAR2CSA ELISA (Table 2). Control ELISA employing scrambled constructs and constructs from other PfEMP1 not implicated in the pathogenesis of PAM were consistently completely negative (data not shown). VAR2CSA constructs produced in *Escherichia coli* cells that should promote disulphide bond formation in secreted proteins (Barfod *et al*., 2006) were also consistently negative in ELISA (data not shown). Each of the VAR2CSA-reactive monoclonal antibodies had absolute specificity for either DBL3-X (PAM2.8, PAM6.1 and PAM8.1; originating from two donors) or DBL5-ε (PAM3.10, PAM4.7, PAM5.2 and PAM7.5; also originating from two donors) (Fig. 3A and Table 2). This pattern of reactivity was confirmed when the monoclonal antibodies were used to detect surface-expressed 3D7-VAR2CSA domains on transfected Jurkat cells in a flow cytometry assay (Fig. 3A). Competition ELISA to examine the epitopes recognized by the DBL3-X and DBL5-ε-reactive monoclonal antibodies showed that the DBL3-X-reactive antibodies PAM2.8 and PAM6.1 targeted antigenically distinct epitopes (Fig. 3B), while two (PAM3.10 and PAM7.5) of the DBL5-ε-reactive antibodies appeared to target neighbouring or overlapping epitopes (Fig. 3C).

**Table 2:** Domain specificity of human VAR2CSA-specific IgG1 monoclonal antibodies, determined by ELISA.

		Monoclonal antibody							
Parasite	Domain	PAM1.4	PAM2.8	PAM3.10	PAM4.7	PAM5.2	PAM6.1	PAM7.5	PAM8.1
3D7	DBL3-X	–	+	–	–	–	+	–	–
	DBL5-ε	–	–	+	–	+	–	+	–
FCR3	DBL3-X	–	+	–	–	–	–	–	+
	DBL5-ε	–	–	+	+	+	–	n.d.	–

n.d., not determined.

### Human VAR2CSA DBL3-X-specific monoclonal IgG antibodies recognize epitopes that vary between parasite isolates

**Fig. 4:**
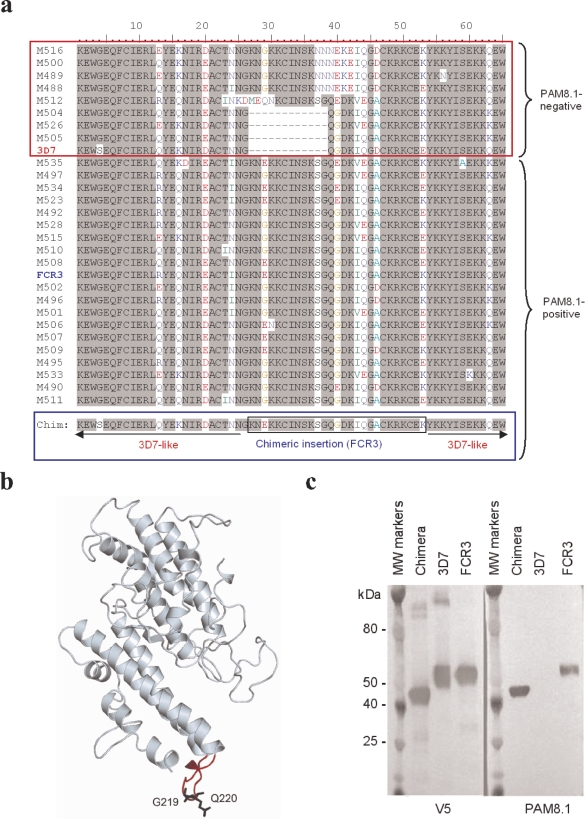
PAM8.1 recognition of VAR2CSA DBL3-X. A. Amino acid sequence in the region of the domain where interclonal variation affected PAM8.1 recognition of *Baculovirus*-produced DBL3-X constructs from 29 genetically distinct *P. falciparum* isolates, including the sequence of a chimeric protein constructed to add PAM8.1 reactivity to the otherwise PAM8.1-negative 3D7 VAR2CSA DBL3-X sequence. B. Structural model of the 3D7 DBL3-X domain. The predicted loop region where parasite isolates recognized by PAM8.1 have a definite insertion compared with 3D7 is shown in red. The 3D7 residues flanking the insert, G1474 and Q1475 (positions 26 and 39 in A), are highlighted in black. C. Western blots of recombinant 3D7- and FCR3-specific VAR2CSA DBL3-X constructs, and of the above-mentioned chimeric construct, probed with loading control antibody V5 (left) and PAM8.1 (right). MW, molecular weight.

The pattern of monoclonal antibody recognition of IEs varied between parasite isolates. This is consistent with the finding that the *var2csa* sequence is composed of conserved stretches separated by stretches with substantial interclonal diversity (Duffy *et al*., 2006a; Trimnell *et al*., 2006), and the prediction that B-cell epitopes in VAR2CSA DBL3-X locate mainly to polymorphic, surface-exposed parts of VAR2CSA (Dahlbäck *et al*., 2006). We therefore cloned and sequenced 43 VAR2CSA DBL3-X domains from placental parasite isolates. A subset of 29 of these domains selected to represent the overall VAR2CSA DBL3-X diversity was expressed as *Baculovirus* recombinant proteins and used in ELISA to test the specificity of the three VAR2CSA DBL3-X-specific monoclonals. The PAM2.8 antibody reacted with 25, PAM6.1 with eight and PAM8.1 with 20 of the domain variants (Fig. 4A). A multiple sequence alignment of all the proteins indicated that the main difference between the PAM8.1-negative and -positive proteins was a C-terminal 16-amino-acid stretch that maps to a polymorphic region of 3D7-VAR2CSA DBL3-X, which is predicted to be a surface-exposed loop (Dahlbäck *et al*., 2006) (Fig. 4B). Residues in this region either were deleted in the PAM8.1-negative proteins or had a different amino acid composition compared with the PAM8.1-positive variants (Fig. 4A). To substantiate this possibility we constructed a chimeric protein where the 16-amino-acid stretch from a PAM8.1-positive domain variant (FCR3) was transferred to the corresponding site in a PAM8.1-negative variant lacking this sequence (3D7) (Fig. 4A, bottom). The recombinant proteins corresponding to the unmodified FCR3 sequence and the chimeric construct both tested positive in Western blots probed with PAM8.1, in contrast to the recombinant protein representing the authentic 3D7 sequence (Fig. 4C), thus confirming the predicted position of the PAM8.1 epitope. It was not possible to predict the exact targets of PAM2.8 and PAM6.1 by multiple alignments of the primary sequences.

### Human monoclonal antibody PAM1.4 effectively selects for expression of VSA_PAM_ and increased transcription of VAR2CSA

**Fig. 5:**
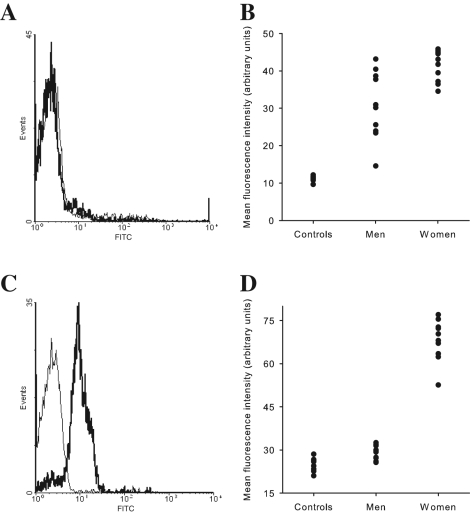
PAM1.4 selection of parasite line EJ27. A. Pre-selection reactivity of monoclonal antibody PAM1.4 (heavy line) and negative control monoclonal antibody (thin line) with the surface of EJ27-IEs. B. Pre-selection non-PAM VSA-type recognition pattern of EJ27 by IgG in plasma from *P. falciparum*-exposed men and women and in plasma from non-exposed adults. C. Reactivity of PAM1.4 antibody (heavy line) and negative control antibody (thin line) with the surface of erythrocytes infected by the EJ27 after a single round of selection for reactivity with PAM1.4. D. Post-selection VSA_PAM_-type recognition pattern of EJ27 by IgG in plasma from *P. falciparum*-exposed men and women and in plasma from non-exposed adults.

PAM1.4 stained VSA_PAM_-expressing IEs, but did not yield any bands in Western blots, and did not react with any of the VAR2CSA constructs when tested in ELISA or by flow cytometry (Tables 1 and 2). These observations are compatible with recognition by this antibody of a conformational epitope in VAR2CSA, but also with recognition of an unidentified non-VAR2CSA PAM-specific IE surface antigen. To address this question, we tested the ability of PAM1.4 to enrich VSA_PAM_-expressing IEs in two parasite lines (EJ24 and EJ27) initially expressing non-PAM-type VSA and only marginally recognized by PAM1.4 (Fig. 5A and B, and data not shown). Although both isolates were originally obtained from the peripheral blood of pregnant women, and thus expected to express VSA_PAM_, isolates expressing non-PAM VSA – such as EJ24 and EJ27 – are occasionally found (Ofori *et al*., 2003, and our unpublished data). Remarkably, a single round of PAM1.4 antibody selection of EJ27 (Fig. 5C and D) and EJ24 (data not shown) resulted in rapid emergence of IEs uniformly recognized by PAM1.4 and expressing VSA_PAM_ (Fig. 5C and D). Quantitative real-time polymerase chain reaction (PCR) analysis of the isolates showed increases in *var2csa* transcription in response to the selection for PAM1.4 reactivity (EJ24: twofold and EJ27: 30-fold). In addition, EJ24 acquired reactivity with the VAR2CSA-specific antibodies PAM2.8, PAM3.10, PAM6.1 and PAM7.5 following selection for PAM1.4 reactivity (Table 1). EJ27 did not acquire additional reactivity following PAM1.4 selection, probably because of interclonal differences in the VAR2CSA epitopes recognized by the other monoclonal antibodies. Taken together, these findings are consistent with VAR2CSA being the antigenic target of PAM1.4.

### Concluding remarks

We have shown that it is possible to interrogate the memory B-cell repertoire of malaria-immune donors to estimate frequencies of *P. falciparum*-specific B cells, and to isolate specific monoclonal antibodies with specificity for the VSA repeatedly implicated as the main targets of acquired protective immunity to malaria. We have used this approach to demonstrate that PAM can result in acquisition of high frequencies of B cells producing IgG with specificity for VSA_PAM_, and in particular VAR2CSA, strengthening previous evidence that these antigen specificities are critically important in acquired protective immunity to PAM. We furthermore show that VSA_PAM_-specific memory B cells acquired in response to PAM primarily target polymorphic, conformation-dependent epitopes that are reproduced by *Baculovirus*-produced recombinant antigen constructs. Our data thus underscore the importance of VAR2CSA in acquired immunity to PAM. However, the findings reported here and elsewhere (Dahlbäck *et al*., 2006) also suggest that *var2csa* diversity (Duffy *et al*., 2006a; Trimnell *et al*., 2006) is driven by protective immunity to PAM, a situation that may complicate development of VAR2CSA-based vaccines against PAM (Beeson *et al*., 2006). IE adhesion to CSA, which is thought to be a critical element in the pathogenesis of PAM (Fried and Duffy, 1996), is mediated by VAR2CSA as documented by recent knockout studies (Viebig *et al*., 2005; Duffy *et al*., 2006b), and several CSA-adhesive domains have been identified in the antigen (Gamain *et al*., 2005). Recent studies in mice suggest that vaccination can elicit broadly reactive antibodies that can block VAR2CSA-dependent IE adhesion to CSA (Gamain *et al*., 2004; Bir *et al*., 2006), but whether such antibodies are ever produced in humans in response to PAM, how clinically relevant they are, and whether they can be induced by vaccination in humans remain unanswered questions. Understandably, present research is highly focused on the identification of functionally constrained epitopes in these domains that are critical for IE adhesion to CSA and of intergenomically conserved epitopes that may serve as targets of antibodies interfering with it. Human monoclonal antibodies appear to be a powerful tool in this research.

## Experimental procedures

### Parasite cultivation and selection for infected erythrocyte surface expression of VSA_PAM_

All *P. falciparum* parasites used in this study were grown in 0^+^ erythrocytes (Cranmer *et al*., 1997). 3D7, FCR3 and NF54 are long-term *in vitro* cultured lines. All expressed non-PAM-type VSA, meaning that intact IEs were recognized to a similar extent by IgG in the plasma of *P. falciparum*-exposed men and sympatric, multigravid women in a flow cytometry assay of VSA expression (Fig. 1, 3D7-SM) (Staalsoe *et al*., 1999). The 3D7 subline 3D7-SM was derived by human plasma antibody selection of 3D7 for expression of non-PAM-type PfEMP1 associated with severe malaria in children as described (Staalsoe *et al*., 2003; Jensen *et al*., 2004). The VSA_PAM_-expressing subline 3D7-BeWo was selected by repeated panning of IEs on the choriocarcinoma line BeWo as described elsewhere (Haase *et al*., 2006). Parasites were considered as expressing VSA_PAM_ if the level of labelling of intact IEs by IgG in a panel of plasma samples from *P. falciparum*-exposed multigravid women was significantly higher than the level in plasma from sympatric men (Fig. 1, FCR3-CSA and NF54-VAR2CSA). The characteristics of the plasma IgG recognition pattern of VSA_PAM_ and non-PAM-type VSA have been documented in detail elsewhere (Ricke *et al*., 2000; Staalsoe *et al*., 2001). Sublines of FCR3 and NF54 (FCR3-CSA and NF54-CSA respectively) were selected for expression of VSA_PAM_ by repeated panning of IEs on CSA *in vitro* (Fried and Duffy, 1996; Ricke *et al*., 2000). NF54-CSA was further selected for IE reactivity with rabbit antiserum specific for VAR2CSA DBL5-ε, resulting in subline NF54-VAR2CSA (Salanti *et al*., 2004). Additional sublines of FCR3 (FCR3-A745 and FCR3-CD36) expressing non-PAM VSA were selected by repeated panning on CSA-negative CHO cells (CHO-A745) and recombinant CD36, respectively, essentially as described for BeWo and CSA selection. Isolates EJ24 and EJ27 were obtained from the peripheral blood of pregnant, *P. falciparum*-exposed women and adapted to *in vitro* culture (Giha *et al*., 1999). Both isolates were selected for expression of VSA reacting with the human VSA_PAM_-specific monoclonal antibody PAM1.4 (see below), essentially as described (Staalsoe *et al*., 2003), but using Protein A-coated magnetic microbeads, as VSA_PAM_-expressing IEs are prone to non-specific labelling by second-step antisera (Creasey *et al*., 2003; Rasti *et al*., 2006).

### Memory B-cell immortalization and cloning

Peripheral blood mononuclear cells (PBMC) from *P. falciparum*-exposed, recently pregnant multiparous women were isolated and cryopreserved as described (Hviid *et al*., 1993). At the day of use, PBMC were thawed and IgG^+^ memory B cells were isolated using CD22 microbeads (Miltenyi) followed by cell sorting as described (Traggiai *et al*., 2004). Cells were immortalized at 100 cells per well in multiple 96-well plates using EBV in the presence of CpG ODN2006 (Microsynth, Switzerland) (Hartmann and Krieg, 2000) and irradiated PBMC as described (Traggiai *et al*., 2004).

### Antibody characterization by flow cytometry, immunofluorescence microscopy and ELISA

Polyclonal B-cell culture supernatants were screened by flow cytometry (Staalsoe *et al*., 1999) for IgG reactivity with the surface of intact, unfixed erythrocytes infected by FCR3-CSA, NF54-VAR2CSA and 3D7-SM. VSA_PAM_-reactive B-cell lines, selected on the basis of their rate of IgG synthesis and growth rates, were cloned by limiting dilution as described (Traggiai *et al*., 2004) and the selectivity of the human monoclonal antibodies produced by the clones for IEs expressing VSA_PAM_ was confirmed as above. The reactivity of the antibodies with the surface of wet-mounted antibody-labelled IEs was further verified by immunofluorescence microscopy, using an LSM5 scanning microscope (Carl Zeiss MicroImaging) (Salanti *et al*., 2004). The IgG subclass of all the human monoclonal antibodies was determined by ELISA and verified by flow cytometry using isotype-specific antibodies (Megnekou *et al*., 2005).

### Antibody characterization by Western blotting

Parasite cultures were enriched for erythrocytes infected by late trophozoite/schizont-stage parasites by exposure to a strong magnetic field (Paul *et al*., 1981; Staalsoe *et al*., 1999). Protein extracts of purified IEs were prepared with 2% SDS in PBS containing complete protease inhibitor (Roche, Basel, Switzerland). The extracts were boiled in denaturing loading buffer and separated in pre-cast tris-acetate 5–8% SDS gradient gels (Invitrogen, Tåstrup, Denmark) with tris-acetate running buffer (Invitrogen), employing pre-stained broad-range molecular weight markers (Bio-Rad, Herlev, Denmark). The separated proteins were transferred to PVDF membranes by wet blotting in transfer buffer containing 20% isopropanol, 20 mM tris-acetate and 0.1% SDS, followed by blocking with 5% skimmed-milk powder in TBS-T buffer. Membranes were incubated with the human monoclonal antibodies or a monoclonal mouse anti-exon 2 antibody, followed by incubation with a secondary anti-human or anti-mouse AP-conjugated antibody (Sigma, MO, USA) and developed using a 5-bromo-4-chloro-3-indoyl phosphate and nitroblue tetrazolium solution (Sigma). *Baculovirus*-produced proteins and a pre-stained ProSieve protein marker (Cambrex) were run on pre-cast 4–12% SDS gradient gels (Invitrogen, Tåstrup, Denmark) with NuPage MOPS SDS running buffer (Invitrogen). Proteins were transferred to a nitrocellulose membrane by wet blotting using a buffer of 20% methanol, 25 mM Tris and 192 mM glycine. Following blocking in 5% skimmed-milk powder in TBS-T buffer, membranes were incubated for 1 h with either a 1:5000 dilution of horseradish peroxidase-conjugated loading control antibody anti-V5 (R960-25, Invitrogen) or a 1:1000 dilution of PAM8.1. The PAM8.1-probed membrane was further incubated with a 1:1000 dilution of a secondary anti-human IgG antibody (P0214, Dako Cytomation). Membranes were developed using 3-amino-9-ethyl-carbazole tablets dissolved in acetone, 50 mM sodium acetate and 30% H_2_O_2_.

### Recombinant VAR2CSA proteins

Regions of re-codonized 3D7-*var2csa* (PFL0030c) and FCR3-*var2csa* covering the entire exon 1 were subcloned into the pBAD-TOPO vector, transferred with the V5 and HIS tag to the pAcGP67-A transfer vector (BD Biosciences), produced as recombinant proteins in *Baculovirus*-infected insect cells, and purified as described (Salanti *et al*., 2004). We have previously shown that *Baculovirus*-produced VAR2CSA constructs are conformationally intact, as they induce production of rabbit antisera reactive with native VAR2CSA on the surface of IEs (Barfod *et al*., 2006). The following regions (indicated by encoded amino acids numbers) were produced: A: 0–446, E: 447–876, I: 877–1208, L: 1209–1572, P: 1573–1980, T: 1981–2355, Z: 2356–2685. In addition, regions of re-codonized 3D7-*var2csa* were cloned into the pDisplay vector (Invitrogen) for surface expression in Jurkat cells (below). The pDisplay vector supplies a signal sequence and a *trans*-membrane domain for surface expression, and two epitope tags (haemagglutinin and c-myc) for monitoring protein expression. The following regions were expressed in Jurkat cells (for details, see below): B: 0–449, C: 0–669, D: 443–870, F: 674–1253, G: 680–906, H: 680–1011, J: 1201–1379, K: 1201–1579, M: 1258–1967, N: 1380–1579, O: 1559–1992, Q: 1776–2131, R: 1965–2666, S: 1981–2336, U: 1981–2524, V: 2147–2666, X: 2210–2666 and Y: 2317–2666. Different variants of DBL3-X were cloned and produced as described (Dahlbäck *et al*., 2006). For the cloning of the chimeric construct composed of 5′ 3D7-VAR2CSA DBL3-X and 3′ FCR3-VAR2CSA DBL3-X, we used the primers 5′: cggaattcGATACAAATGGTGCCTGT and 3′: CATTTCTTTTCATTCTTACCATTATTAGTGCA to generate the 3D7-specific, and 5′: AAGAATGAAAAGAAATGTATTAATTC and 3′: atttgcggccgcATATACTGCTATAATCTCC to generate the FCR3-specific part of the chimera. These primers amplify a slightly smaller PCR product than the original primers used for making the FCR3 and 3D7 DBL3-X constructs, and this is reflected in the smaller molecular size of the chimeric construct. The two PCR products were gel-purified and used in a second PCR using the two outer primers to generate a PCR product consisting of 5′ 3D7 and 3′ FCR3, with an EcoRI site and a NotI site. The PCR product was cloned into a modified pAcGP67-A vector (BD Biosciences) and expressed in insect cells as described (Salanti *et al*., 2004).

### ELISA

VSA_PAM_-reactive monoclonal IgG-containing supernatants were tested in ELISA (Dodoo *et al*., 2000) for reactivity with the recombinant VAR2CSA proteins produced in *Baculovirus*-infected insect cells. In addition, the epitope specificities of monoclonal antibodies targeting DBL3-X (PAM2.8 and PAM6.1) and DBL5-ε (PAM3.10, PAM5.2 and PAM7.5) were analysed by competition ELISA. PAM3.10 and PAM6.1 were purified on ÄktaXpress (GE Healthcare, Brøndby, Denmark) using a HiTrap Protein G HP 1 ml column with subsequent desalting on a HiPrep 26/10 desalting column (GE Healthcare). Purified IgG was biotinylated using EZ-link maleimide-PEO solid phase as described by the manufacturer (Pierce, Bonn, Germany). Microtitre plates (Nunc, Roskilde, Denmark) were coated with recombinant DBL3-X (8.3 μg ml^−1^) or DBL5-ε (10.4 μg ml^−1^) in PBS (1 h, 37°C). After blocking of the plates, biotinylated PAM3.1-specific IgG (0.72 μg ml^−1^) or biotinylated PAM6.1-specific IgG (2.5 μg ml^−1^) and increasing concentrations of the competitor monoclonal culture supernatants were added to triplicate wells. PAM1.4 (unknown VSA_PAM_-specificity) and PAM2.8 (DBL3-X-specific) were added as negative controls to DBL3-X-coated and DBL5-ε-coated plates respectively. Bound biotinylated IgG was detected by incubation (1 h, room temperature) of wells with horseradish peroxidase-conjugated streptavidin (1 μg ml^−1^, 100 μl well^−1^; Pierce, Bonn, Germany).

### Analysis of monoclonal IgG specificity by flow cytometry of *var2csa*-transfected Jurkat cells

The human T-cell line Jurkat (Gillis and Watson, 1980) was cultured in RPMI 1640, supplemented with 25 mM HEPES and l-glutamine (Gibco, Tåstrup, Denmark), 10% FCS, 100 IU ml^−1^ penicillin and 100 μg ml^−1^ streptomycin. Two million cells were seeded into each well of a six-well plate and transfected with 3–4 μg of plasmid DNA (see above) and 4 μl of DMRIE-C transfection reagent (Invitrogen) according to the manufacturer's instructions. Within 48 h of transfection, the cells were washed and re-suspended at 1 × 10^6^ ml^−1^ in PBS supplemented with 2% FCS. Cells (1 × 10^5^) were incubated with human monoclonal antibodies or with a haemagglutinin mouse antibody for 30 min followed by two washes and labelling by secondary FITC-conjugated anti-human IgG or anti-mouse IgG antibody. Flow cytometry analysis was essentially as above.

### Monoclonal antibody epitope mapping by recombinant VAR2CSA DBL3-X constructs and *in silico* modelling

Recombinant VAR2CSA DBL3-X constructs from 29 genotypically distinct *P. falciparum* isolates were produced in *Baculovirus*-infected insect cells and tested in ELISA essentially as described above. The three-dimensional structure of the 3D7-VAR2CSA DBL3-X sequence (PFL0030c, amino acids 1217–1559) was modelled *in silico* as described elsewhere (Dahlbäck *et al*., 2006). Briefly, the crystal structure of EBA-175 F1 (PDB code 1ZRO chain A) (Tolia *et al*., 2005), which has 28% sequence identity to the 3D7-VAR2CSA DBL3-X domain, was used as a template. The model was evaluated with respect to locations of conserved cysteine bridges and buried hydrophobic residues in the structures of DBL domains from EBA-175 F1 and F2 (Tolia *et al*., 2005) and Pk-α DBL (Singh *et al*., 2005).

### Quantitative real-time PCR

Quantitative real-time PCR was performed on cDNA from unselected and PAM1.4 antibody-selected isolates EJ24 and EJ27 using a Rotorgene thermal cycler system (Corbett Research, Cambridge, UK) and a primer set specific for a highly conserved part of the *var2csa* DBL-4ε domain, targeting all *var2csa* genes without bias (Salanti *et al*., 2003; Tuikue Ndam *et al*., 2005). Selection-induced changes in *var2csa* transcription were quantified as described (Salanti *et al*., 2003).
